# Systemic and Renal Dynamics of Free Sulfhydryl Groups during Living Donor Kidney Transplantation

**DOI:** 10.3390/ijms23179789

**Published:** 2022-08-29

**Authors:** Nora A. Spraakman, Annemieke M. Coester, Arno R. Bourgonje, Vincent B. Nieuwenhuijs, Jan-Stephan F. Sanders, Henri G. D. Leuvenink, Harry van Goor, Gertrude J. Nieuwenhuijs-Moeke

**Affiliations:** 1 Department of Anesthesiology, University of Groningen, University Medical Centre Groningen, Hanzeplein 1, 9713 GZ Groningen, The Netherlands; 2 Department of Surgery, Amphia Hospital, Molengracht 21, 4818 CK Breda, The Netherlands; 3 Department of Gastroenterology and Hepatology, University of Groningen, University Medical Centre Groningen, Hanzeplein 1, 9713 GZ Groningen, The Netherlands; 4 Department of Surgery, Isala Zwolle, Dr. Van Heesweg 2, 8025 AB Zwolle, The Netherlands; 5 Department of Internal Medicine, Division of Nephrology, University Medical Center Groningen, Hanzeplein 1, 9713 GZ Groningen, The Netherlands; 6 Department of Surgery, University of Groningen, University Medical Centre Groningen, Hanzeplein 1, 9713 GZ Groningen, The Netherlands; 7 Department of Pathology and Medical Biology, University of Groningen, University Medical Centre Groningen, Hanzeplein 1, 9713 GZ Groningen, The Netherlands

**Keywords:** ischemia–reperfusion injury, oxidative stress, free thiols, redox, kidney transplantation

## Abstract

During ischemia–reperfusion injury (IRI), reactive oxygen species are produced that can be scavenged by free sulfhydryl groups (R-SH, free thiols). In this study, we hypothesized that R-SH levels decrease as a consequence of renal IRI and that R-SH levels reflect post-transplant graft function. Systemic venous, arterial, renal venous, and urinary samples were collected in donors and recipients before, during, and after transplantation. R-SH was measured colorimetrically. Systemic arterial R-SH levels in recipients increased significantly up to 30 sec after reperfusion (*p* < 0.001). In contrast, renal venous R-SH levels significantly decreased at 5 and 10 min compared to 30 sec after reperfusion (both *p* < 0.001). This resulted in a significant decrease in delta R-SH (defined as the difference between renal venous and systemic arterial R-SH levels) till 30 sec after reperfusion (*p* < 0.001), indicating a net decrease in R-SH levels across the transplanted kidney. Overall, these results suggest trans-renal oxidative stress as a consequence of IRI during kidney transplantation, reflected by systemic and renal changes in R-SH levels in transplant recipients.

## 1. Introduction

Post-operative kidney graft assessment is currently based on clinical scores and post-transplant biopsies with the histological assessment [1,2]. These approaches have their limitations since clinical signs often lag behind the renal injury, and a kidney biopsy is an invasive procedure and associated with complications. Therefore, there is an urgent need for non-invasive early graft markers of damage, especially since, nowadays, increasing numbers of lower quality kidneys of extended criteria donor (ECD) kidneys are used. In order to develop these assessment tools and potential subsequent therapeutic strategies, more insight into graft injury and repair is needed. One of the most important mechanisms affecting the viability of the graft is ischemia and reperfusion injury (IRI) [3,4,5]. In the early post-transplantation period, IRI will reveal itself as delayed graft function (DGF) or primary non-function (PNF) [4]. In addition, the impact of IRI in driving cellular injury and rejection in kidney transplantation is increasingly recognized [6]. In the long term, IRI may lead to fibrosis that may subsequently result in allograft dysfunction [7].

IRI consists of complex pathophysiology in which numerous injurious molecular and cellular cascades are involved [5]. Mitochondrial injury most likely plays an important initiating step in IRI. During reperfusion, mitochondria are the major source of reactive oxygen species (ROS) through the generation of superoxide by reverse action of complex I of the electron transport chain due to succinate accumulation [8]. The massive amount of mitochondrially produced ROS is a critical factor in IRI and ROS are directly or indirectly responsible for oxidative damage to cells and tissues via lipid peroxidation and protein carbonylation [8,9,10]. As a consequence, disruption of adenosine triphosphate formation, dysregulation of calcium levels, and induction and opening of mitochondrial permeability transition pores occurs [9,10,11]. The latter leads to the release of mitochondrial substances such as cytochrome C, succinate, and mitochondrial DNA into the cytosol. These substances can act as damage-associated molecular patterns and are able to activate various cellular processes such as activation of the immune system or induce apoptosis [11,12]. This injurious cascade with increased ROS formation as a key factor is one of the main mechanisms linked to suboptimal kidney function post-transplantation [13].

The intracellular redox potential, a measure of the intracellular oxidizing capacity of the cell, is a highly regulated process imperative for normal physiological functioning. Under physiological circumstances, the redox potential is slightly negative compared to the extracellular environment [14]. Oxidative stress induces a change in the cellular redox potential with a shift towards a more positive potential, which causes the formation of disulfide bridges through oxidation of free sulfhydryl groups (R-SH, free thiols). This mechanism reveals that R-SHs are the transducers of these redox-regulated events by oxidation of ROS via electron uptake [15,16]. As such, R-SHs are protective against oxidative stress as they potently scavenge ROS and constitute active components of the antioxidant machinery [17]. R-SH are present in both cells and in extracellular fluids and are mainly embedded in proteins (particularly albumin, based on its high abundance and its transporting capacity of low-molecular-weight (LMW) thiols), whereas they also occur as LMW thiols, e.g., homocysteine, cysteine, and glutathione [15,18]. Therefore, the level of R-SH may reflect current redox status and may be regarded as an indirect read-out for ROS generation, with high levels reflecting a favorable whole-body redox status and decreased levels reflecting increased interaction with ROS and, thus, an unfavorable redox status [19].

To gain insight into the course of ROS generation and the relation to post-transplantation graft function, we measured R-SH before, during, and after transplantation in multiple sample types in a unique cohort of living donor kidney transplantation (LDKT) donor-recipient couples. In this study, we hypothesized that R-SH levels decrease as a consequence of renal IRI and that R-SH levels reflect post-transplant graft function.

## 2. Results

### 2.1. Baseline Characteristics

Baseline characteristics of donors and recipients are summarized in Table 1. Donors (*n* = 24) had musculoskeletal and cardiovascular (e.g., hypertension) as most common co-morbidity (45.8% and 33.3% respectively). The mean age was 52.8 (±11.6) years, and 45.8% were male. Mean body mass index (BMI) was 27 (±3.2) kg/m^2^. Nine (37.5%) donors were active smokers. Medications used by donors were mainly proton pump inhibitors (PPIs, 12.5%), statins (16.7%), and antihypertensive (25%) drugs. The median pre-donation measured glomerular filtration rate (mGFR) was 112.5 (96.3–128.8) ml/min. The mean age of recipients (*n* = 24) was 52.3 (±11.7) years, and 37.5% were male. Cardiovascular comorbidities were most common (66.7%). Recipients used various medications, with PPIs (50%), statins (45.8%), and antihypertensive (87.5%) being the most common. Eleven (45.8%) received a kidney from an unrelated donor, and 33.3% were transplanted pre-emptively. First warm ischemia time (WIT1), defined as the time between clamping of the renal artery and cold perfusion with University of Wisconsin (UW) solution, was 4 (3–4) min. Cold ischemia time (CIT), defined as cold storage time, was 177 (155–207.5) min, and second warm ischemia time (WIT2), defined as the time between cold storage and reperfusion (anastomosis time), was 42.1 (±6.7) min. DGF occurred in one (4.2%) recipient. Median estimated glomerular filtration rate (eGFR) measurements were stable over time up to 24 months post-transplantation. An episode of acute rejection in a 2-year follow-up occurred in four recipients (16.7%). All rejections were T-cell mediated. One (4.2%) recipient died on day nine post-transplantation due to cardiac arrest.

### 2.2. Plasma R-SH in Different Sample Types 

**Figure 1:**
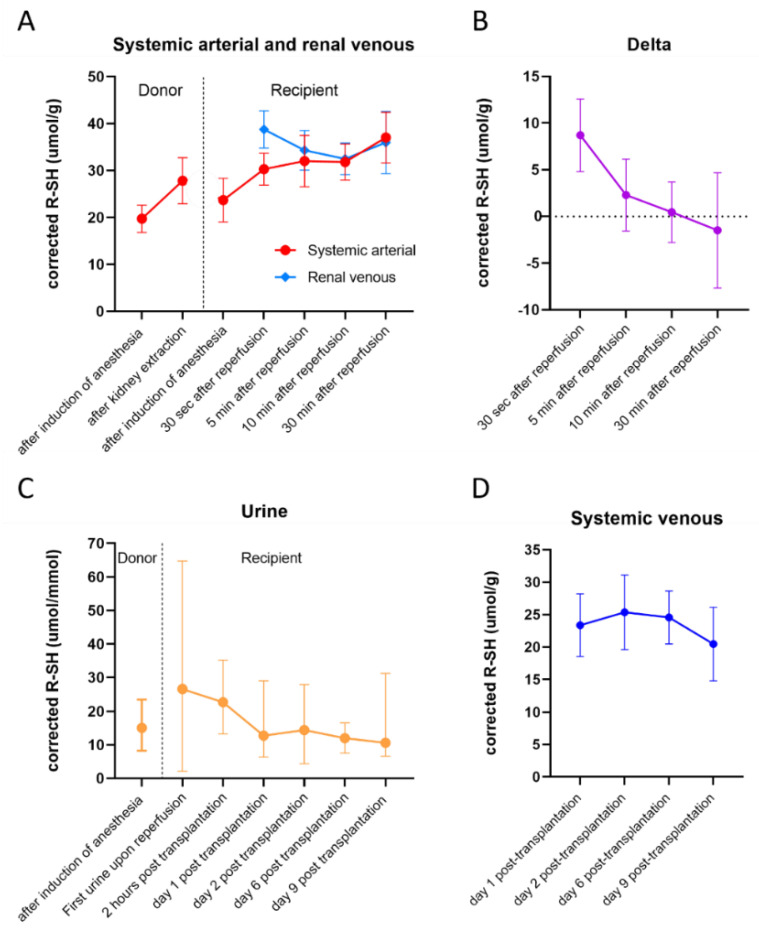
R-SH levels in samples types in donors and recipients. Data given as mean (±SD) or median (IQR). R-SH is corrected by plasma albumin levels or urinary creatinine levels. (**A**): Mean systemic arterial (µmol/g) and mean renal venous R-SH (µmol/g) in donors and recipients. (**B**): Mean delta (renal venous-systemic arterial) R-SH (µmol/g) in recipients. (**C**): Median urinary R-SH (µmol/mmol) in donors and recipients. (**D**): Median systemic venous R-SH (µmol/g) in recipients. *Abbreviations: R-SH: free sulfhydryl groups*.

Plasma R-SHs in different sample types at different time points are displayed in Figure 1. In the donors, mean systemic arterial R-SH was significantly higher after kidney extraction (27.8 (±4.9) µmol/g of albumin) compared to after induction of anesthesia (19.7 (±2.9) µmol/g of albumin, *p* < 0.001, Figure 1A). In the recipients, mean R-SH increased significantly after reperfusion compared to after induction of anesthesia (23.4 (±4.6) µmol/g of albumin, all *p* < 0.001). During reperfusion, a plateau phase was observed between 30 sec and 10 min after reperfusion. At 30 min after reperfusion (37.0 (±5.4) µmol/g of albumin), a significant increase was observed when compared to 30 sec (30.3 (±3.4) µmol/g of albumin, *p* < 0.001), 5 min (32.0 (±5.5) µmol/g of albumin, *p* < 0.001) and 10 min after reperfusion (31.8 (±3.8) µmol/g of albumin, *p* < 0.001). Furthermore, recipients had significantly higher R-SH after induction of anesthesia than donors at that same time point (*p* < 0.002). Over time, systemic arterial R-SH in the recipients did significantly change (*p* < 0.001) (Table 2 and Table 3). Renal venous R-SH significantly decreased from 38.8 (±4.0) µmol/g at 30 sec after reperfusion to 34.3 (±4.2) µmol/g at 5 min (*p* < 0.001) and to 32.5 (±3.4) µmol/g at 10 min after reperfusion (*p* < 0.001, Figure 1A). Overall, the dynamics of renal venous R-SH in the recipients significantly changed after reperfusion (*p* < 0.001) (Table 2 and Table 3). In order to evaluate the usage or addition of R-SH by the kidney itself, the trans-renal (Delta; renal venous—systemic arterial) dynamics of R-SH were analyzed (Figure 1B). Delta R-SH significantly decreased from 30 sec after reperfusion (8.7 (±3.9) µmol/g) to 5 min (2.3 (±3.8) µmol/g, *p* < 0.001), 10 min (0.5 (±3.2) µmol/g, *p* < 0.001) and 30 min after reperfusion (−1.5 (±6.2) µmol/g, *p* < 0.001). The mean delta was negative at 30 min after reperfusion, indicating a net decrease in renal venous R-SH compared to systemic arterial R-SH. Overall, delta R-SH in the recipients significantly changed after reperfusion (*p* < 0.001) (Table 2 and Table 3). Post-transplantation R-SH of recipients at different time points are displayed in Figure 1D. In the recipient, median systemic venous R-SH at day nine post-transplantation (20.7 (18.8–22.2) µmol/g) was significantly lower compared to day one (22.8 (20.1–25.2) µmol/g, *p* = 0.007), day two (24.8 (21.2–27.9) µmol/g, *p* = 0.020) and day six post-transplantation (25.4 (22.7–26.8) µmol/g, *p* = 0.017). Over time, systemic venous R-SH in the recipients did significantly change during the post-transplantation period when time was entered as covariate (*p* = 0.012 and *p* = 0.004) (Table 2) and as factor (*p* = 0.009) (Table 3).

## 3. Discussion

The aim of this post hoc analysis was to study the dynamics of R-SH levels in this unique cohort of LDKT donor-recipient couples to gain insight into the course of ROS generation and the relation with post-transplantation graft function. We observed significant changes in R-SH across timepoints in all sample types. Our study indicates marked oxidative stress in the transplanted kidney upon reperfusion, as demonstrated by a significantly decreasing delta R-SH which reflects a net trans-renal decrease in R-SH levels. Systemic arterial R-SH significantly increased over time with simultaneously a significant decrease in renal venous R-SH up to 30 min after reperfusion. Systemic venous and urinary R-SH showed a similar pattern, namely a significant decrease in R-SH in the post-transplantation phase.

To the best of our knowledge, this is the first study that reports on the trans-renal dynamics of oxidative stress in LDKT recipients. Systemic arterial R-SH levels increased up to 30 min after reperfusion. A similar increase in R-SH following an ischemic event has previously been observed in a study by Abdulle et al. in which, after cold-induced vasoconstriction in the hands, systemic venous R-SH levels increased after the restoration of blood flow, which was defined as a ‘thiol rebound’ [20]. Although the change of R-SH concentrations over time differed between healthy controls, patients with primary Raynaud’s phenomenon, and patients with systemic sclerosis in that study, a net increase in R-SH levels was observed in all of these groups suggesting a tightly regulated physiological response [20]. Thioredoxins, constituting disulfide-reducing proteins, could explain this physiological increase in R-SH. Thioredoxin is a potent deglutathionylating protein that is able to deglutathionylate proteins in the presence of high levels of oxidized glutathione, such as in conditions of oxidative stress [21]. This possibly indicates an acute phase response to IRI and possibly to surgery by increasing R-SH availability over a period of time [14]. Similarly, our study showed an overall net significant post-transplantation decrease in systemic venous R-SH. This may support the acute phase response after reperfusion, after which R-SH levels ‘normalizes’ as IRI in the kidney and recipient is expected to subside.

Levels of renal venous R-SH concentrations at 30 sec after reperfusion are remarkably at a higher level compared to systemic arterial levels at that same time point. Literature on the dynamics of R-SH within the kidney itself, and thus on R-SH measured in renal venous samples, is scarce. However, several suggestions could explain this observation. First of all, the kidney itself may increase R-SH availability, and after this initial boost of R-SH, the kidney is ‘depleted’ of R-SH. However, due to the preservation of the kidney in a cold environment and the consequent hypometabolism, we do not expect a large contribution of the kidney to increase R-SH availability. Another explanation of kidney R-SH release could be the role of the glycocalyx during IRI [22]. Glycocalyx consists of plasma albumin, and destruction due to IRI leads to the release of, among others, albumin. As explained before, albumin accounts for a large share of the R-SH pool, and this could also explain this phenomenon. Moreover, the kidneys are flushed with UW-solution. UW-solution contains glutathione [23]. After this initial flush, it is likely that a certain amount of UW-solution remains in the kidney and is expelled during the reperfusion period, which may elucidate renal venous R-SH levels during reperfusion. Fourth, we should consider that the systemic arterial and renal venous compartments differ in terms of volume but also in function. Systemic arterial R-SH may already react with ROS released from other organs and tissues, as it is likely that there is a systemic stress response during IRI and surgery in general, whereas renal venous R-SH represents a more local stress response.

Systemic arterial R-SH and renal venous R-SH changed significantly over time, culminating in a net decrease or ‘consumption’ of R-SH across the transplanted kidney, reflecting trans-renal oxidative stress after reperfusion. This is shown by a decreasing ‘delta’ R-SH (defined as the difference between renal venous and systemic arterial R-SH levels) after reperfusion. This decreasing delta, with a negative delta at 30 min after reperfusion, may suggest that there is an increased consumption of R-SH over the kidney, likely due to the increased production of ROS. This is supported by the fact that the reperfusion period during IRI is mainly responsible for the large amounts of ROS production due to the restoration of normoxemia. Upon reperfusion, the succinate that accumulated during ischemia is oxidized and creates an environment favoring the reverse action of the electron transport chain. This leads to large amounts of ROS formation and a compromised redox balance [8,11]. Our results support this phenomenon since we demonstrated a reduction of delta R-SH levels between renal venous and arterial samples.

Urinary R-SH levels were relatively low compared to plasma levels. Urinary R-SH levels in the first urine upon reperfusion as well as 2 h post-transplantation were significantly higher compared to day six post-transplantation. This is in line with the systemic arterial increase observed during reperfusion. Furthermore, this also could suggest that the filtration of R-SH might be linked to oxidative stress, as we observe a significant decrease at day six post-transplantation. Urinary R-SHs were not corrected for albumin since albumin and other high-molecular-weight plasma proteins are usually not filtrated in the presence of an intact glomerular filtration barrier. Therefore, we consider that the urinary R-SH pool will primarily constitute non-protein-bound free thiols. However, existing knowledge on the composition of the free thiol pool in human urine is currently insufficient to accurately define the relative contributions of relevant redox compounds and their specific roles in renal physiology. In addition, the process of filtration of R-SH and tubular reabsorption or secretion by the kidney is largely unknown. Previous research by Aebi et al. showed that glutathione and cysteine are increasingly excreted after intravenous infusion of glutathione. This is in line with our suggestion that LMW thiols can be excreted in urine upon the increase in plasma concentration [24]. To the best of our knowledge, literature about the excretion of free thiols is limited, and further research is needed.

Our study also sheds light on the fact that the timing of R-SH measurement needs to be taken into consideration. Studies measuring R-SH at multiple time points in the setting of kidney transplantation are limited [25]. Recently, Nielsen et al. studied plasma R-SH levels in relation to early and one-year graft function. They measured at baseline, 30 and 90 min after reperfusion, and day one, day five, and 12 months post-transplantation in plasma of deceased donor kidney transplantation recipients. Nielsen et al. reported a significant positive association between R-SH levels on day one and day five with mGFR at day five and 12 months post-transplantation. In contrast, it was demonstrated that in patients experiencing DGF, R-SH at 30 min and 90 min were significantly higher. Levels at these timepoints might reflect the extent of the injury and ROS formation. However, levels of R-SH over time were not reported and discussed, which complicates the comparison of the results of our study. In addition, no clarification can be given on the type of blood samples that were taken during this study [25]. As we demonstrated in our results, the type and timing of sampling are crucial, as we observed distinct concentrations of R-SH over time and between sample types. It is imperative to reconsider sample type and timing before implementing the use of R-SH levels as a marker of oxidative stress in research and clinical care. 

Strengths of our study include the presence of sequential measurements over time, which started during organ procurement in the donor and ended at day nine post-transplantation. This provides valuable insight into the dynamics of systemic R-SH levels over time. Furthermore, we measured R-SH in different body fluids (using systemic venous, renal venous, arterial, and urinary samples) and observed differences in quantities and time course. Especially R-SH measured in the plasma directly obtained from the reperfused kidney showed intriguing results with a decreasing delta (renal venous–systemic arterial) R-SH up to 30 min after reperfusion as an indicator of net trans-renal oxidative stress readily after reperfusion. In addition, the fact that splint urine samples were taken ensured R-SH originated from the transplanted kidney only.

A few limitations of this study also must be addressed. This post hoc analysis of the VAPOR-1 study consisted of a small cohort of 24 patients receiving a high-quality kidney with low incidences of inferior post-transplantation outcomes due to limited IRI during LDKT. This could also explain the absence of associations between R-SH and suboptimal graft and patient outcome parameters. We did not observe significant associations between R-SH levels and kidney outcomes. Nielsen et al. studied a larger patient population with recipients of deceased donor kidneys [25]. Deceased donor kidneys endure more extensive oxidative stress, and this likely explains the difference in results when compared to our study. Second, we were not able to determine levels of additional oxidative stress biomarkers as there was not sufficient sample availability to measure additional redox parameters. Finally, our assay is not able to discriminate between specific types of free thiols. Non-protein-bound antioxidant compounds such as glutathione, homocysteine, or cysteine, comprise LMW thiols and are of minor importance to the antioxidant capacity, as the greatest share is represented by protein-bound free thiols (60–75%). Thus far, existing knowledge is insufficient to define the relative contributions of all these individual compounds and their specific roles in redox signaling pathways [17,18]. Therefore, Ellman’s reagent was chosen as the technique to provide results about the whole-body redox status, with an optimized protocol produced by our research group.

In conclusion, this is the first study that shows the dynamics of R-SH in LDKT donors and recipients. We showed a decrease in delta R-SH levels till 30 min after reperfusion, indicating increased ROS formation as a consequence of IRI. R-SH measurement has the potential to be a non-invasive, easily measured biomarker of oxidative stress. Hence, future research with larger cohorts needs to be conducted to study the interaction of R-SH groups and ROS while taking the timing of sample collection and sample type into careful consideration. Therefore we will proceed with studying this interaction in a large cohort of recipients of deceased donor kidneys participating in the VAPOR-2 study (NCT02727296).

## 4. Materials and Methods

### 4.1. The Study Design and Population

This study is a post hoc analysis of the VAPOR-1 (Volatile Anesthetic Protection of Renal Transplants-1) trial, a prospective randomized controlled trial on the effect of two different anesthetic agents (propofol vs. sevoflurane) on graft and patient outcome in LDKT [26]. The VAPOR-1 trial was approved by the local Institutional Review Board (METc 2009/334), registered with ClinicalTrials.gov (NCT01248871), and conducted at the University Medical Centre of Groningen between September 2010 and October 2014. Details of the study have been published previously [26]. A total of 60 donor-recipient couples (120 patients in total) met the inclusion criteria, gave written informed consent, and were randomly assigned to one of the following groups: PROP; propofol for donor and recipient, SEVO; sevoflurane for donor and recipient, PROSE; propofol for donor and sevoflurane for recipient. Three couples were excluded from the primary analysis due to violation of the surgical or immunosuppressive protocol, leaving 57 donor-recipient couples for analysis. For the present analysis, couples were pooled into one group, of which 24 couples were selected based on sample availability and cost feasibility.

### 4.2. Clinical Parameters

Baseline donor and recipient characteristics were collected from VAPOR-1 trial database. Characteristics included demographics, medical history, medication use, and health status. Perioperative data included ischemia times, human leukocyte antigens mismatches, and positive panel reactive antibodies. Outcome parameters are occurrence of DGF, acute rejection episodes, graft loss, and patient mortality. Pre-donation mGFR in donors was assessed with use of iodine 125-iothalamate, performed at least 3 months before donation. eGFR in recipients was calculated with the use of the Chronic Kidney Disease Epidemiology Collaboration (CKD-EPI) formula at months 1, 3, 6, 12, and 24 post-transplantation.

### 4.3. Sample Points and Sample Measurements

**Figure 2:**
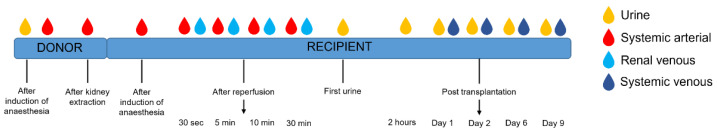
Timeline sample collection in donor and recipient.

Blood and urine samples were taken at standardized time points listed in Figure 2. Blood (EDTA) samples were obtained from both the systemic venous and systemic arterial circulation. In addition, samples were obtained from the renal venous circulation of the reperfused kidney with use of a sampling catheter placed in the renal/gonadal vein of the transplanted kidney. Urine samples were obtained from a splint catheter inserted in the ureter of the transplanted kidney and exteriorized as a suprapubic catheter. This splint was removed on day 10 [26]. Samples were stored at −80 °C until R-SH measurement. Samples were diluted 1:4 with 0.1 M Tris buffer (pH 8.2). After measuring background absorption at 412 nm using a Sunrise microplate reader (Tecan Group AG, Männedorf, Switzerland), with a reference measurement at 630 nm, 10 μL 3.8 mM 5,5′-Dithio-bis(2-nitrobenzoic acid) (DTNB; Ellman’s Reagent, CAS-number 69-78-3, Sigma Aldrich Corporation, St. Louis, MO, USA) in 0.1 M phosphate buffer (pH 7) was added to the samples; after an incubation period of 20 min at room temperature sample absorbances were measured again. Concentrations of free thiol groups were determined by parallel measurements of an L-Cysteine (CAS-number 52-90-4, Fluka Biochemika, Buchs, Switzerland) standard curve [15.625 mM to 1000 mM] in 0.1 M Tris/10 M EDTA (pH 8.2). The variability of the R-SH measurements in the samples had a coefficient of variation (CV) < 4. R-SH measurements in plasma were corrected by plasma albumin, as albumin accounts for a large share of the R-SH pool in blood both quantitatively and qualitatively [18]. Albumin was measured using Albumin (Bromocresol green) colorimetric assay Kit (Abcam, ab235628) according to the manufacturer’s instructions. Samples were diluted 1:50 in the assay buffer before analysis and read at absorbance 620 nm using a Spectromax microplate reader. Urinary R-SH was corrected for urine dilution by urinary creatinine, which measured according to standard protocol. 

### 4.4. Statistical Analysis

For statistical analysis, SPSS version 23 (IBM Corp, Armonk, NY, USA) and GraphPad Prism version 8.4.2 (GraphPad software, Inc., La Jolla, CA, USA) were used. Prior to analysis, an inter-plate intensity normalization procedure was performed using the plate median as normalization factor. This normalization procedure adjusts the data by equalizing the median value for each plate to the median for that of all the other plates. Continuous variables were tested for normal distribution with use of the Shapiro–Wilk normality test and visualized by normal probability (Q-Q) plots. Descriptive statistics were presented as mean (±standard deviation (SD)) for normally distributed variables, median (interquartile range (IQR)) in case of non-normal distributions or proportions n with corresponding percentages (%) for categorical variables. Depending on normality, paired t-tests or Wilcoxon signed-rank tests were used to analyze between time points. Linear mixed models were used to assess changes in R-SH over time, where time was entered as a fixed effect; either as covariate (to analyze the linear trend) or as factor (to analyze each time point against baseline). When time was entered as covariate, we tested polynomials to see which model best describes the change over time. Estimates of covariates are presented as b with standard error (SE) including 95% confidence interval (CI). As time was entered as a factor, estimated means were calculated with corresponding 95% confidence intervals (CIs). Effects for baseline characteristics (based on literature), subject grouping and time points were tested to improve the model. Best-fitted models are presented based on the log-likelihood statistics. Depending on normality distribution, Pearson’s or Spearman’s correlation coefficients were used for analyzing associations between R-SH and kidney outcome parameters. False discovery rate (FDR, according to the Benjamini–Hochberg procedure) of 5% was used to correct for multiple testing. Statistical significance was set at *p*-value ≤ 0.05 for all comparisons.

## Figures and Tables

**Table 1:** Baseline demographic and clinical characteristics.

**Donor**	***n* = 24**
Age [y]	52.8 (±11.6)
Male [*n* (%)]	11 (45.8%)
BMI [kg/m^2^]	27 (± 3.2)
Smoking [*n* (%)]	9 (37.5%)
Cardiovascular risk factors [*n* (%)]	8 (33.3%)
Pre-donation mGFR [mL/min/1,73 m^2^]	112.5 (96.3–128.8)
**Recipient**	***n* = 24**
Age [y]	52.3 (±11.7)
Male [*n* (%)]	9 (37.5%)
BMI [kg/m^2^]	25.5 (±4.0)
Cardiovascular risk factors [*n* (%)]	16 (66.7%)
Unrelated donor [*n* (%)]	11 (45.8%)
Pre-emptive transplantation [*n* (%)]	8 (33.3%)
Re-transplantation [*n* (%)]	3 (12.5%)
≥3 Human leukocytes antigen mismatches [*n* (%)]	12 (50%)
Positive panel reactive antibodies (≥15%) [*n* (%)]	4 (16.7%)
WIT1 [min] CIT [min] WIT2 [min]	4 (3–4) 177 (155–207.5) 42.1 (±6.7)
**Post-transplantation Outcomes**	*n* = 24 *
DGF [*n* (%)]	1 (4.2%)
eGFR 1 month post-transplantation [mL/min/1,73 m^2^]	47.5 (41.3–59.5)
eGFR 3 months post-transplantation [mL/min/1,73 m^2^]	43.1 (37.4–58.2)
eGFR 6 months post-transplantation [mL/min/1,73 m^2^]	48.5 (38.9–61.5)
eGFR 12 month post-transplantation [mL/min/1,73 m^2^]	48.4 (38.5–54.4)
eGFR 24 month post-transplantation [mL/min/1,73 m^2^]	50.3 (40.2–61.8)
Acute rejection 2 years [*n* (%)]	4 (16.7%)
Graft loss [*n* (%)]	0 (0%)
Mortality [*n* (%)]	1 (4.2%)

Data given as mean (±SD), median (IQR) or n (%). * Post-transplantation outcomes were collected for all 24 recipients, excluding graft functions outcomes of 1 recipient (died at day nine post-transplantation due to cardiac arrest). *Abbreviations: BMI: body mass index; mGFR: measured glomerular filtration rate, WIT1: warm ischemia time 1; CIT: cold ischemia time; WIT2: warm ischemia time 2, DGF: delayed graft function, eGFR: estimated glomerular filtration rate.*

**Table 2:** Results of linear mixed models: time as covariate.

	*p*-Value	b	SE b	95% CI
**Systemic arterial R-SH**				
Time	<0.001 *	24.86	3.72	17.4–32.3
Time2	<0.001 *	−8.04	1.38	−10.8–−5.3
Time3	<0.001 *	0.86	0.15	0.6–1.2
**Renal venous R-SH**				
Time	<0.001 *	−11.1	1.56	−14.3–−7.9
Time2	<0.001 *	2.0	0.30	1.4–2.6
**Delta R-SH**				
Time	<0.001 *	−9.2	1.6	−12.6–−5.9
Time2	<0.001 *	1.3	0.3	0.6–1.9
**Systemic venous R-SH**				
Time	0.012 *	6.6	2.6	1.5–11.8
Time2	0.004 *	−1.5	0.5	−2.5–−0.5
**Urinary R-SH**				
Time	0.156	-5.3	**3.7**	−12.6–−2.0

Linear mixed models were used to assess changes in R-SH over time, where time was entered as covariate. Best-fitted models are presented with estimate of fixed effect (b with SE, including 95% CI). Statistical significance (*) was set at *p*-value ≤ 0.05. *Abbreviations: R-SH: Free sulfhydryl groups, b: estimate of fixed effect; SE: standard error; CI: confidence interval.*

**Table 3:** Results of linear mixed models: time as factor.

	Estimated Means (95% CI)	F-Statistic	*p*-Value
**Systemic arterial R-SH**		28.9	<0.001 *
After induction of anesthesia	23.7 (21.9–25.5)		
30 sec after reperfusion	30.2 (28.2–32.1)		
5 min after reperfusion	31.8 (29.8–33.9)		
10 min after reperfusion	31.8 (29.8–33.9)		
30 min after reperfusion	37.0 (34.9–39.0)		
**Renal venous R-SH**		12.2	<0.001 *
30 sec after reperfusion	38.8 (36.7–40.8)		
5 min after reperfusion	34.0 (31.9–36.1)		
10 min after reperfusion	32.3 (30.2–34.5)		
30 min after reperfusion	35.6 (33.4–37.8)		
**Delta R-SH**		20.9	<0.001 *
30 sec after reperfusion	8.7 (6.8–10.6)		
5 min after reperfusion	2.3 (0.3–4.2)		
10 min after reperfusion	0.4 ((−1.6)–2.4)		
30 min after reperfusion	−1.5 ((−3.6)–0.6)		
**Systemic venous R-SH**		4.2	0.009 *
Day 1 post-transplantation	23.4 (21.3–25.4)		
Day 2 post-transplantation	25.4 (23.3–27.4)		
Day 6 post-transplantation	24.6 (22.5–26.6)		
Day 9 post-transplantation	20.5 (18.3–22.6)		
**Urinary R-SH**		1.34	0.252
First urine upon reperfusion	55.5 (26.6–84.7)		
2 h post-transplantation	26.2 ((−4.3)–56.6)		
Day 1 post-transplantation	19.0 ((−12.2)–50.2)		
Day 2 post-transplantation	52.1 (20.8–83.3)		
Day 6 post-transplantation	16.4 ((−14.8)–47.7)		
Day 9 post-transplantation	18.8 ((−11.7)–49.3)		

Linear mixed models were used to assess changes in R-SH over time, where time was entered as a factor. As time was a categorical variable, estimated means were calculated with corresponding 95% confidence intervals (CIs). Statistical significance (*) was set at *p*-value ≤ 0.05. *Abbreviations: R-SH: Free sulfhydryl groups.*

## Data Availability

The data that support the findings of this study are available from the corresponding author upon reasonable request.

## Supplementary Materials

The following supporting information can be downloaded at: https://www.mdpi.com/article/10.3390/ijms23179789/s1.
