# Supplementary material for: Systemic and Renal Dynamics of Free Sulfhydryl Groups during Living Donor Kidney Transplantation
Source: Int J Mol Sci. 2022 Aug 29;23(17):9789. doi: 10.3390/ijms23179789 (PMC9455962; doi:10.3390/ijms23179789)
Supplement: Supplementary file 1 [file ijms-23-09789-s001.zip › ijms-1852519-supplementary.pdf]

Table S1 Relation of R-SH to outcome

	eGFR 1-month	eGFR 3-month	eGFR 6-month	eGFR 12-month	eGFR 24-month	Acute rejection episodes
**Systemic arterial R-SH**						
Donor						
After induction of anesthesia	R 0.244<br>P 0.274	R 0.264<br>P 0.235	R 0.274<br>P 0.218	R 0.091<br>P 0.688	R 0.124<br>P 0.583	R 0.163<br>P 0.458
After kidney extraction	R -0.239<br>P 0.285	R -0.008<br>P 0.971	R -0.043<br>P 0.850	R -0.051<br>P 0.822	R -0.030<br>P 0.894	R 0.009<br>P 0.968
Recipient						
After induction of anesthesia	R 0.335<br>P 0.118	R 0.640<br>P 0.001	R 0.530<br>P 0.009	R 0.420<br>P 0.046	R 0.442<br>P 0.035	R -0.109<br>P 0.611
30 sec after reperfusion	R 0.025<br>P 0.916	R 0.379<br>P 0.090	R 0.282<br>P 0.216	R 0.198<br>P 0.389	R 0.240<br>P 0.294	R -0.348<br>P 0.122
5 min after reperfusion	R 0.203<br>P 0.404	R 0.612<br>P 0.005	R 0.576<br>P 0.010	R 0.342<br>P 0.152	R 0.313<br>P 0.193	R -0.470<br>P 0.042
10 min after reperfusion	R 0.492<br>P 0.038	R 0.673<br>P 0.002	R 0.546<br>P 0.019	R 0.434<br>P 0.072	R 0.464<br>P 0.052	R -0.136<br>P 0.590
30 min after reperfusion	R 0.164<br>P 0.502	R 0.445<br>P 0.056	R 0.580<br>P 0.009	R 0.256<br>P 0.290	R 0.211<br>P 0.385	R -0.084<br>P 0.726
**Renal venous R-SH**						
30 sec after reperfusion	R 0.323<br>P 0.154	R 0.564<br>P 0.008	R 0.372<br>P 0.097	R 0.355<br>P 0.115	R 0.315<br>P 0.165	R -0.084<br>P 0.726
5 min after reperfusion	R 0.033<br>P 0.849	R 0.535<br>P 0.018	R 0.403<br>P 0.087	R 0.261<br>P 0.280	R 0.258<br>P 0.287	R -0.247<br>P 0.280
10 min after reperfusion	R 0.167<br>P 0.494	R 0.537<br>P 0.018	R 0.392<br>P 0.097	R 0.230<br>P 0.343	R 0.278<br>P 0.249	R -0.250<br>P 0.301
30 min after reperfusion	R -0.105<br>P 0.689	R 0.040<br>P 0.878	R 0.087<br>P 0.740	R -0.209<br>P 0.421	R 0.007<br>P 0.978	R -0.344<br>P 0.149
**Delta R-SH**						
30 sec after reperfusion	R 0.229<br>P 0.331	R 0.207<br>P 0.381	R 0.090<br>P 0.705	R 0.104<br>P 0.662	R -0.084<br>P 0.726	R 0.289<br>P 0.216
5 min after reperfusion	R -0.253<br>P 0.296	R -0.284<br>P 0.239	R -0.377<br>P 0.111	R -0.199<br>P 0.413	R -0.161<br>P 0.509	R 0.438<br>P 0.060
10 min after reperfusion	R -0.352<br>P 0.151	R -0.236<br>P 0.347	R -0.198<br>P 0.430	R -0.199<br>P 0.430	R -0.232<br>P 0.354	R -0.341<br>P 0.166
30 min after reperfusion	R -0.299<br>P 0.244	R -0.246<br>P 0.342	R -0.397<br>P 0.097	R -0.418<br>P 0.095	R -0.225<br>P 0.385	R -0.075<br>P 0.776
**Systemic venous R-SH**						
Day 1 post transplantation	R 0.158<br>P 0.471	R -0.086<br>P 0.698	R 0.144<br>P 0.511	R 0.081<br>P 0.713	R 0.278<br>P 0.199	R -0.492<br>P 0.015
Day 2 post transplantation	R 0.027<br>P 0.904	R 0.200<br>P 0.360	R 0.147<br>P 0.503	R 0.028<br>P 0.900	R -0.127<br>P 0.564	R -0.163<br>P 0.447
Day 6 post transplantation	R 0.269<br>P 0.214	R 0.084<br>P 0.704	R 0.059<br>P 0.790	R 0.144<br>P 0.511	R 0.144<br>P 0.512	R -0.084<br>P 0.696
Day 9 post transplantation	R 0.211<br>P 0.347	R 0.068<br>P 0.763	R 0.172<br>P 0.443	R 0.053<br>P 0.816	R 0.111<br>P 0.622	R -0.297<br>P 0.179
**Urinary R-SH**						
Donor						
after induction of anesthesia	R -0.062<br>P 0.779	R -0.200<br>P 0.361	R -0.323<br>P 0.133	R -0.443<br>P 0.034	R -0.436<br>P 0.037	R 0.057<br>P 0.791
Recipient						
First urine upon reperfusion	R 0.052<br>P 0.815	R 0.110<br>P 0.618	R -0.049<br>P 0.825	R 0.035<br>P 0.875	R 0.134<br>P 0.542	R -0.007<br>P 0.975
2 hours post transplantation	R -0.144<br>P 0.535	R -0.301<br>P 0.185	R -0.177<br>P 0.444	R -0.087<br>P 0.708	R -0.086<br>P 0.711	R 0.248<br>P 0.265
Day 1 post transplantation	R -0.257<br>P 0.261	R -0.127<br>P 0.584	R -0.040<br>P 0.862	R -0.137<br>P 0.555	R -0.245<br>P 0.284	R -0.161<br>P 0.486
Day 2 post transplantation	R -0.021<br>P 0.930	R -0.190<br>P 0.423	R -0.272<br>P 0.247	R -0.190<br>P 0.422	R 0.035<br>P 0.885	R -0.290<br>P 0.202
Day 6 post transplantation	R -0.167<br>P 0.480	R -0.311<br>P 0.182	R -0.362<br>P 0.117	R -0.224<br>P 0.342	R -0.241<br>P 0.307	R 0.214<br>P 0.351
Day 9 post transplantation	R 0.276<br>P 0.213	R 0.321<br>P 0.145	R 0.084<br>P 0.709	R 0.416<br>P 0.054	R 0.364<br>P 0.095	R 0.028<br>P 0.902

Depending on normality distribution, Pearson's or Spearman's correlation coefficients were used to analyse relation between R-SH and kidney outcome parameters. False discovery rate (FDR, according to the Benjamini-Hochberg procedure) of 5% was used to correct for multiple testing. Statistical significance (*) was set at P-value ≤ 0.05 for all comparisons. *Abbreviations: R-SH: Free sulfhydryl groups*
